# Measuring competition in primary care—Evidence from Sweden

**DOI:** 10.1371/journal.pone.0304994

**Published:** 2024-07-15

**Authors:** Sofie Vengberg, Mio Fredriksson, Ulrika Winblad, David Isaksson

**Affiliations:** Health Services Research, Department of Public Health and Caring Sciences, Uppsala University, Uppsala, Sweden; Abu Dhabi University, UNITED ARAB EMIRATES

## Abstract

**Introduction:**

In many tax-based healthcare systems, policymakers have introduced reforms that promote provider competition with the intention of improving the quality and efficiency. Healthcare competition is usually defined spatially, with local markets often being identified as a circle around each provider. We argue that existing local market definitions can be improved to better capture actual local markets. For pro-competition reforms to potentially lead to the gains envisioned by policymakers, a crucial condition is the actual emergence of competitive markets. However, limited research has been conducted on competition in primary care markets, despite primary care constituting a vital part of a healthcare system.

**Aim:**

The study aims to contribute to the debate on how to define local markets geographically and to examine provider competition in Swedish primary care.

**Methods:**

A cross-sectional study was conducted using data on all individuals and all primary care providers in Sweden. Local markets were defined as: fixed radius (1 km and 3 km); variable radius; and variable shape—our new local market definition that allows markets to vary in both size and shape. Competition was measured using the Herfindahl-Hirschman index and a count of the number of competitors within the local market.

**Results:**

Fixed radius markets fail to capture variation within and across geographical areas. The variable radius and variable shape markets are similar but do not always identify the same competitors or level of competition. Furthermore, competition levels vary significantly in Swedish primary care. Many providers operate in monopoly markets, whereas others face high competition.

**Conclusions:**

While the variable shape approach has the potential to better capture actual markets and more accurately identify competitors, further analyses are needed. Moreover, Swedish policymakers are advised to decide whether to still pursue competition and if so, take measures to improve local market conditions in monopolies.

## 1. Introduction

A strong primary care sector is often described as a vital part of any well-functioning healthcare system, contributing to better cost control as well as having beneficial effects on health outcomes and population health [1–3]. At the same time, primary care sectors around the world face several common challenges related to financial constraints, changing demands, and technological advancements. In many countries, measures have been sought to reform primary care with the aim of improving responsiveness and strengthening the sector [4]. To enhance provider responsiveness to patients and improve overall provider performance, policymakers have introduced healthcare reforms that promote provider competition within tax-based healthcare systems.

When analysing healthcare competition, it is crucial, regardless of the specific healthcare sector studied, to acknowledge its spatial dimension. The spatial dimension is evident as providers compete foremost with other providers located nearby, and as patients mainly seek healthcare through a physical visit to a nearby healthcare provider. Distance is also a main predictor of choice of healthcare provider, especially in primary care [5–7]. Therefore, provider competition in healthcare settings is measured spatially in local markets. Local markets are often defined by a radius drawn from each provider—either a fixed radius, applied to all providers, or a variable radius determined by the distance between a provider and its patients [8]. As markets in reality are not naturally confined to perfect circles, we believe that variable definitions of local market areas can be refined to better capture actual markets. Our aim is therefore to contribute to the debate on how to define local markets geographically, by creating a new way to define local markets and analysing it in relation to existing local market definitions.

Pro-competition reforms can be seen as comprehensive toolboxes consisting of policies targeting the supply side by steering providers’ revenues and incentives (e.g., through design of payments to providers), but where demand side policies ensuring patient choice are essential [9]. Economic theory states that competition in markets with fixed prices positively affects provider performance, with healthcare providers in more competitive markets being more likely to improve their performance than those less exposed to competition [10]. While there is an overall consensus in the theoretical literature on the positive effects of competition in price-regulated healthcare markets (although refinements of the theoretical model have been suggested) [10–12], empirical findings are more mixed [13–17]. Despite the central role of primary care in any healthcare system, primary care markets have been studied far less than hospital and nursing markets [18]. Since context matters when studying healthcare markets, empirical findings are not easily transferred to other countries or sectors. It can be argued that the conditions for a competitive market are better in primary care sectors than in hospital sectors because a larger number of small sized providers operate in primary care sectors. The fact that primary care practices are much smaller than hospitals potentially means both lower entry costs and fewer concerns about economies of scale [19]. Still, the empirical literature on primary care competition in settings with regulated prices is limited, although growing in recent years [20–27]. So far, both positive effects [22]—albeit sometimes limited [20]—as well as negative [27] and mixed effects [25] have been found.

When studying a pro-competition reform, it is, however, not only relevant to analyse whether provider competition improves provider and market performance, but also whether a competitive market structure in fact has emerged. In this paper, we will address the latter aspect in a primary care setting. In general, primary care competition is still a largely under-researched area. While there are some studies on the effects of competition [20–27], little descriptive information is—with some exceptions [22, 24]—presented on the level of competition in primary care market. Results from empirical studies indicate that levels of provider competition are higher in primary care settings [22, 24] than in hospital settings [e.g. 28]. However, there is considerable variation in how different primary care sectors are organized, making generalizations difficult. Therefore, in addition to contributing to the debate on local market definitions, we also aim to empirically examine competition in Swedish primary care. In Sweden, patients can choose primary care provider, reimbursements to all primary care providers are based on patient choices, and entry barriers to the primary care market have been decreased over the last decades—features which were introduced or reinforced by the Patient Choice reform in 2010 [25, 29].

In this paper, a previously unexplored methodological approach to delineating local markets is presented and a previously under-researched area is empirically examined. The latter is achieved by analysing a dataset covering all primary healthcare centres and all people residing in Sweden. The study aim is twofold: to contribute to the debate on how to define local markets geographically; and to examine provider competition in Swedish primary care.

## 2. Measuring competition in healthcare markets

Distance to providers is an important predictor of patients’ choice of provider: patients usually seek care through a physical visit to a nearby healthcare provider [5–7, 20]. While the importance of geography has been challenged lately by the increase in telemedicine and digital consultations in primary care [30], this aspect remains important. Distance—both between providers and between providers and their patients—still affects the level of competition providers face and predicts patients’ choice of provider [5, 6].

One distinctive characteristic of healthcare markets is that the providers engage in spatial competition. Competition in healthcare markets is therefore usually depicted through spatial models, describing the highest level of competition near the geographic boundaries of markets, where the cost of switching is lowest for patients [31]. Thus, healthcare markets are generally defined at the local level. When studying spatial competition, there are two central aspects to take into consideration: how to define the local market areas geographically and how to measure the intensity of competition within local markets [8, 32]. These aspects are addressed below.

### 2.1 Approaches to defining local markets

Definitions of local healthcare markets generally fall into three categories: 1) administrative borders, 2) fixed radius, and 3) variable radius. The first category refers to administrative areas such as postcodes, municipalities and counties [33]. For example, Lower Layer Super Output Areas (small geographical areas used in the UK for place-based statistics) have been used to study English general practitioner (GP) markets [6], and counties to study German outpatient practitioners’ markets [21].

While administrative borders provide an easily accessible unit, the market definition has several flaws. First, administrative units are considered unsuitable in settings where patients are not restricted to providers within their administrative area [8]. Second, since administrative market definitions can be used when micro data are unavailable, aggregated market data are often used in the analyses. Consequently, the results might suffer from estimation issues, as data on an aggregate level may not reveal correct information on the provider level [20, 34].

An alternative strategy is to use coordinate data on the location of the providers and draw a circle around each provider to delineate the local market borders [33]. This can be done with a fixed radius applied to all providers or a variable radius set specifically for each provider. For example, a fixed radius of 15 km has been used to define hospital markets in England [13] and a 2 km radius to define English GP markets [20, 26]. A fixed radius is easily computed since only coordinate data on the provider level are needed, but it is important to consider carefully the distance at which the radius is drawn as it will later affect the level of competition [33]. One problem with the fixed radius definition, however, is that it leads to an urban-rural bias, likely overestimating competition in urban areas and under-estimating it in less densely populated areas [8].

Both the administrative border and the fixed radius definition have been criticised for measuring potential rather than actual provider competition [32]. In 1993, Phibbs and Robinson [35] presented a new definition of local hospital markets—a radius market definition that varies depending on the distance between the hospital and its patients, capturing a set percentage of each provider’s nearest patients. Empirically, it has been most widely used in studies of US hospital markets, where the radius has often been set to capture either 75 per cent or 90 per cent of discharged patients residing closest to each provider [35]. In an English setting, hospital markets have been defined based on GP practices’ 95 per cent nearest patients, aggregated to the hospital level [36]. We have not found any studies on primary care markets that use variable radiuses based on the distance between primary care providers and their patients. Brunt et al. [22], however, vary the radiuses depending on population density when examining physician practice competition under Medicare in the US.

The main advantage of the variable radius definition is that it better reflects actual market areas and is able to capture urban-rural differences [8]. A drawback is that it requires patient-level coordinate data and is therefore not always possible for researchers to calculate. The variable radius approach has also received criticism. The main criticism is the risk of endogeneity problems when studying the effect of competition on provider performance. Since high-quality providers are more likely to attract patients from further away than competing low-quality providers, their local markets will likely cover larger areas; thus, they will be exposed to higher competition, leading to potential reversed causality [8, 37]. One way to address this is to conduct sensitivity analyses where the variable radius definition is complemented by other market definitions [36, 37]. Another way is to derive the radius distance for each provider not from patients’ actual choices but from their predicted choices [38].

Additionally, the variable approach could be improved by varying local markets not only in size but also in shape. Variable radius as a way to define the local market has not changed much since its introduction three decades ago [35], despite technological development that enables more refined spatial analyses. Both fixed and variable radius markets provide an inadequate representation of actual markets, which are not naturally confined to perfect circles. In reality, local markets can be divided by topographical and infrastructural features, such as rivers, highways or metro stations [39]. Providers in socioeconomically divergent areas might also have a limited overlap of patients despite being geographically close, resulting in two separate local markets. Therefore, a new definition of local markets that better represents consumers’ behaviour and providers’ catchment areas could offer a valuable contribution to the scientific discussion on how to define local markets. In the methods section, we present the variable shape definition, which allows local markets to vary in both shape and size.

### 2.2 Approaches to measuring competition

Researchers often use the Herfindahl-Hirschman index (HHI) to measure the underlying structure of the market. The HHI, which is not specific to healthcare markets, is in fact a measure of market concentration—i.e. a lack of competition. More precisely, it is the sum of the squared market share (*s*_*i*_) of each competing provider (*i*) in a defined market with *N* providers:

$$HHI=\sum_{i=1}^{N}s_{i}^{2}$$ (1)

It is most common to define competing providers (*i*) as providers located within a local market, but there are examples where providers whose local markets overlap with the local market in question are also included [13, 14]. The HHI is bounded between 0 and 1, where 1 indicates a monopoly and a value close to 0 indicates a low level of market concentration, i.e. a high level of competition.

The main advantage of the HHI is that it considers not only the number of providers in a market but also their size. However, there is a risk of endogeneity when it is used to study the effect of competition on provider performance, as high-quality providers might have attained a larger market share than their competitors [8]. One way to address such an endogeneity problem is to use an HHI based on predicted patient choice rather than actual patient choice [23, 37], as suggested by Kessler and McClellan [38]. When doing so, patient choices are first predicted based on a choice model with provider and patient characteristics and distances between them, and the HHI is subsequently calculated based on these predicted patient choices. The need to use predicted patient choices can be debated as it has been found to be highly correlated with competition measures based on actual patient choice [36]. Another common approach is to include a count of the competitors in the market, often interpreted as the number of providers located in a local market, as an alternative competition measure. The advantage of the count measure is that it is considered exogenous; its drawback is that it lacks information on providers’ size.

Other examples of competition measures can also be found in the literature. For example, Schaumans [24] and Stroka-Wetsch et al. [21] use provider density—i.e., providers per capita—as the main measure of competition among Belgian GPs and German outpatient practitioners, respectively. In a Norwegian study of GP competition, Godager et al. [40] use the number of rival GPs that accepted new patients as the main competition measure. Scott et al. [27], on the other hand, operationalise competition among Australian GPs as distance to competitors.

Outside the primary care literature, the patient flow method has been used to measure competition. With the patient flow method, provider competition is calculated in two steps. First the level of competition is calculated at the neighbourhood level (e.g., at the postcode level), e.g., through an HHI. Second, the level of competition each provider faces is calculated as the weighted mean value of competition intensity in all neighbourhoods that the provider serves [41]. However, patient registration data in primary care settings differ from discharge/admission hospital data used for calculating patient flow models in hospital markets. Not every person registered with a primary care provider is a patient, and when moving, it is not certain that the choice of provider is reconsidered. Measuring primary care competition at neighbourhood level thus risks containing a lot noise.

In summary, all types of local market definitions and competition measures have different advantages and disadvantages. It is important for researchers to be aware of this when deciding on measures and when interpreting the results, since these decisions will affect the empirical findings.

## 4. Materials and methods

### 4.1 Study setting: Swedish primary care

The 21 politically governed regions are at the core of the tax-funded Swedish healthcare system as they are responsible for funding and providing healthcare [42]. In 2007–2009, Patient Choice reforms were implemented in eight of the regions’ primary care sectors [43]. In 2010, a national Patient Choice reform made it mandatory for all regions to create a system where 1) patients can choose primary care provider, 2) primary care providers are reimbursed on the basis of patient choices, and 3) providers are free to establish in any location as long as they fulfil general, regional requirements. The stated objectives of the national Patient Choice reform were to empower patients, increase provider diversity, and stimulate competition among providers, which in turn was anticipated to contribute to improved quality and accessibility [44, 45].

Swedish primary care is provided by publicly funded, multi-disciplinary team practices, referred to as both primary healthcare centres and providers in this paper. The regions reimburse the primary healthcare centres, whereas healthcare professionals are salaried employees. A primary healthcare centre has, on average, four employed general practitioners as well as district nurses, midwives, physiotherapists, psychologists, and other professionals [46]. On average, a healthcare centre employs 20 people [47] and has 8,280 people on the patient list (SD 3,304, median 7,834). In our data from 2016, there are 1,161 primary care providers operating in Sweden, of which 485 (42%) are privately run. A majority of the private healthcare centres are owned by large, for-profit companies [48].

Some regional variations are worth noting. First, each region designs their own the reimbursement system and consequently reimbursement systems vary across regions, but risk-adjusted capitation constitutes the bulk payment in all regions. Second, regional requirements on providers regarding scope of services vary. In general, however, regions require providers to offer a comprehensive range of services, including, for example, family medicine, child healthcare, rehabilitation services, visits to nursing homes, and podiatry [49]. Third, there are some regional differences concerning patient’s options when choosing provider. While all individuals residing in Sweden can chose to register with any primary healthcare centre in the country and change provider twice a year, in some regions, it is also possible for residents to register with a specific physician at the chosen healthcare centre [29, 50, 51]. In most regions, individuals who do not make an active choice of provider are automatically registered with a nearby provider.

The implementation of the Patient Act (46) in 2015 introduced a legal possibility for patients to choose a provider in any region and enabled a rapid emergence of direct-to-consumer telemedicine services, directed to patients across the country (9). Since direct-to-consumer telemedicine is not spatially dependent, this new form of primary healthcare service challenges the spatial feature of provider competition in Swedish primary healthcare. However, this development has taken place since the data for our study were gathered.

### 4.2 Design and data

The study is cross-sectional and includes the total population of individuals residing in Sweden on 31 December 2015, and all primary care providers operating in Sweden in 2016. Coordinate data on individuals’ place of residence, in the coordinate system SWEREF 99 TM, were retrieved from Statistics Sweden’s geographical database (*Geografidatabasen*) on 22 June 2017. To ensure individuals’ anonymity, Statistics Sweden replaced personal identification numbers (*personnummer*) with serial numbers. Additionally, the authors did not have access to exact coordinates as accessed coordinates were placed in the centroid of 250-metre squares by Statistics Sweden. For those residing in more rural areas (~17%), coordinates were placed in 1,000-metre squares. Data on which provider each individual was registered with were sent by each of the 21 regions to Statistics Sweden, which replaced personal identification numbers (*personnummer*) with serial numbers before the authors could access the data on 25 November 2016. Provider addresses were retrieved from the providers’ websites by the authors and assigned coordinates in the coordinate system SWEREF 99 TM. Thereafter, the authors linked the provider-level data to the individual-level data with provider names as identifiers using Stata (v. 16.1).

The combined dataset was then cleaned; observations with missing coordinate and/or registration data and duplicates were removed. The final dataset used in the analyses contained 9,834,197 individual-level observations and 1,161 providers. The individual-level data used in this paper, retrieved from Statistics Sweden and the 21 Swedish regions, contain sensitive information and are protected by confidentiality. Provider-level competition data, containing information on the HHI and the number of providers for each local market definition, can be found in S1 Data.

### 4.3 Strategy for measuring competition

#### 4.3.1 Defining local markets

Since there is debate about the best local market definition, several definitions are included—two existing, frequently used definitions, fixed radius and variable radius, and one new market definition, named variable shape.

Two market sizes are used for the fixed radius definition. To decide on radius distances, the distances between each individual and their provider were calculated and rank ordered. The 10 per cent furthest away from their provider were excluded in order to remove inaccurate data (e.g., individuals who had moved to another part of the country without de-listing from their old provider). For the providers’ 90 per cent nearest residing patients, the mean distance was 1.4 km and the median distance was 2.7 km. Based on this information, radius distances of 1 km and 3 km were chosen. To simplify computations, the market areas were not exact circles but hexadecagons (16-gons).

For the variable radius market, the distances between each provider and its registered individuals were rank-ordered, and the distance needed to capture the 55 per cent nearest residing individuals was identified for each provider. This distance was then used as the radius when circle-shaped markets were drawn around each provider.

**Fig 1:**
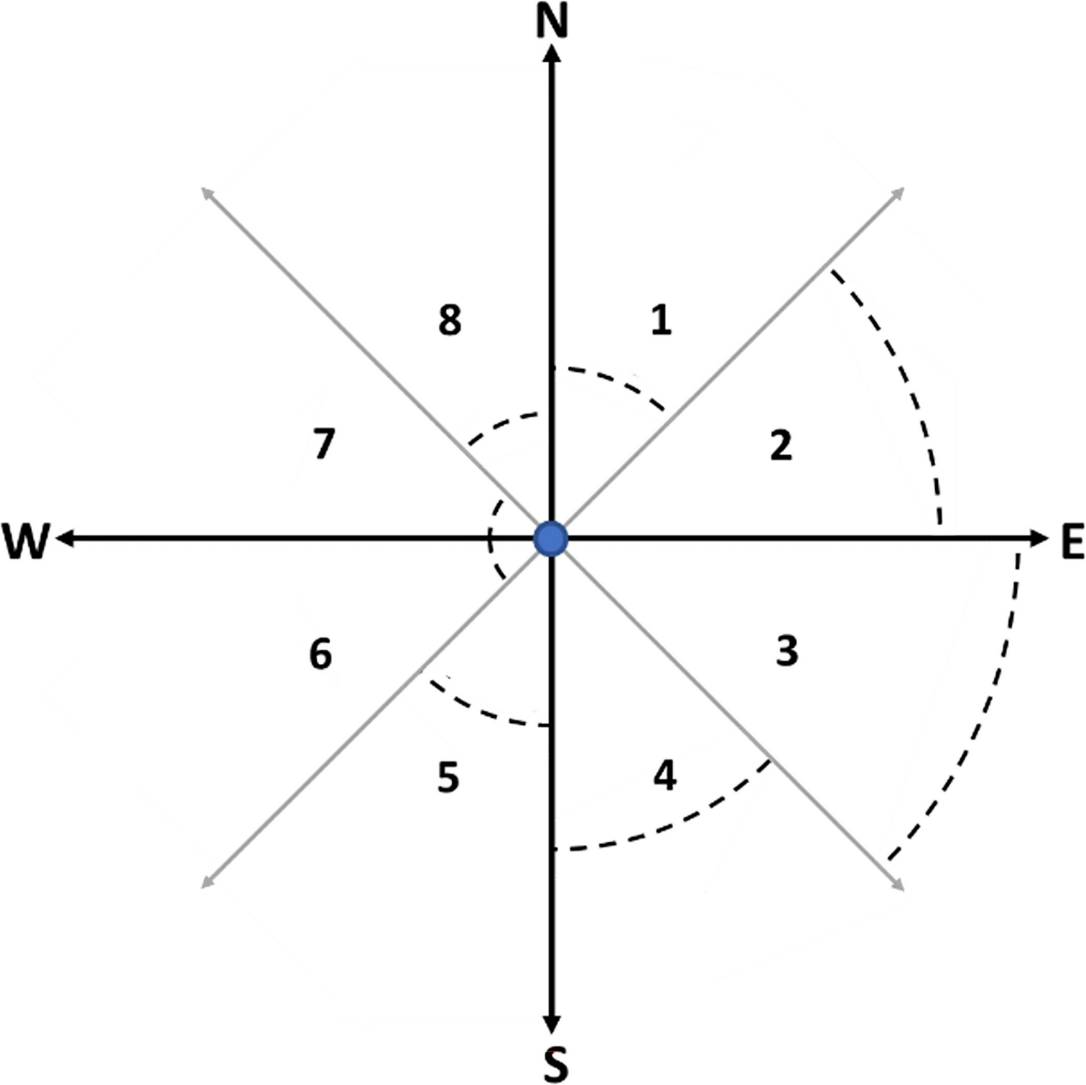
Compass showing step 2 of the variable shape construction. Each number in the figure represents one direction category. Dashed lines visualise how the 70^th^ percentile can vary between categories. The blue dot represents the provider.

The variable shape markets, which vary in both size and shape, were constructed in two main steps. First, the distances between each provider and its registered individuals were rank-ordered, and a subset containing the 80 per cent nearest registered individuals for each provider was created. The purpose of this was to remove inaccurate data and outliers. Second, polygons were created for each provider with stronger weight being given to locations with higher densities of registered individuals. To achieve this, individuals were categorised according to the direction from their chosen provider in which they resided. For example, an individual living north to northeast of their provider was coded in category 1 (see Fig 1). For each direction category, the distances between each provider and its registered individuals were rank-ordered, and the 70 per cent nearest individuals were kept. Since the number of and distances to registered individuals living in each category differ across direction categories, the 70^th^ percentile can result in different distances for each direction category (as exemplified with the dashed lines in Fig 1). Thereafter, the provider coordinates were added to the individual coordinates. The R package *concaveman* [v. 1.1.0; 52] was used to create a variable shape market area for each provider, calculating a concave polygon delimited by all coordinates for each provider. Alpha hulls were restricted to a concavity level of 3, and the length threshold argument on *concaveman* was set to 100. We decided to use these values as they provided a reasonable balance between overly complex polygons and too simple polygons—which could include large areas without any patients registered with the provider as well as lakes and uninhabited areas. Finally, a 10-metre buffer area was added around each polygon to ensure that the border coordinates were included in the market area.

The percentages of nearest patients to include in the two variable market definitions need to be addressed. The decision to base the variable radius markets on the distance for the nearest 55 per cent of patients was to enable comparisons with the variable shape market definition, which was constructed first based on the nearest 80 per cent (step 1) and then on the nearest 70 per cent of registered individuals (step 2). Since 0.8 multiplied by 0.7 equals 0.56, we decided on the 55^th^ percentile for the variable radius market. The percentages chosen for the variable shape market were based on face validity: we studied how the shape and size of polygons changed visually for six primary care providers in four cities of different sizes when the percentiles were changed. The six providers were located in different types of areas, all of which the authors were well acquainted with. This means that lower percentages were chosen in this study compared to studies of hospital competition. We argue that it is reasonable to choose lower percentiles when constructing variable radius markets in primary care than in hospital settings. Patient registration data differ from discharge data, which are often used to identify patients in studies of hospital markets, in that individuals registered with a provider are not necessarily patients. A person who rarely or never uses primary care services might not think of re-registering with another provider when moving. Some individuals’ current address might therefore not reflect providers’ actual catchment areas. In addition, some inaccuracies in data are to be expected in our study due to time lags between patient registration data and individual coordinate data. For instance, people who have moved and changed provider recently might be registered at the old address but at the new provider in our data. There could also be outliers—for example, weekly commuters might register with a provider where they work rather than where they are residing according to official records.

#### 4.3.2 Measuring competition

Two competition measures were calculated for each market definition: the HHI and the number of competitors. The HHI was treated as the main competition measure. Competitors were defined as all providers located in a local market. An alternative HHI based on markets where competitors were defined as those with overlapping local markets—a definition which has been used in studies of English hospital markets [e.g. 13]—is included in S1 Appendix (Table 2). To attain the HHI values, individuals and providers located in each local market were first identified. Thereafter, market shares were calculated for each provider in each local market. For these calculations, individuals who lived in a local market but were registered with a provider outside of the local market were excluded. The HHI was then calculated as the sum of squared market shares of each competing provider in each market. The second competition measure was a count measure of the number of competitors in each local market.

### 4.4 Statistical analyses

The competition measures for each local market definition were examined with descriptive statistics. To examine the extent to which the different market definitions measured the same thing, a Pearson’s correlation test was performed on their respective HHI measures. In addition, spatial point pattern analyses were conducted on a selection of providers to examine the different market definitions visually in relation to where the providers’ patients reside. For this, density rasters (heatmaps) of the location of the selected providers’ 80 per cent nearest registered patients were created using the quartic kernel density estimation function, with the bandwidth of the kernel set to 1,000. The selected providers’ local market polygons were layered on the heatmaps to enable comparison.

Choropleth maps were created to visualise the structure of Swedish primary care market. Choropleth maps of Sweden were used to display descriptive statistics on the HHI, aggregated to the regional level. Maps of all local markets in the two largest regions, Stockholm and Västra Götaland, were also created.

All statistical analyses were performed using R [v. 4.1.1; 53]. R packages *sf* [v. 1.0.3; 54], *sp* [v. 1.4.2; 55, 56], and *concaveman* [v. 1.1.0; 52] were used to construct local markets, while *tmap* [v. 3.3.2; 57] was used to create choropleth maps. QGIS (v. 3.22.4) was used to construct heatmaps.

### 4.5 Ethical considerations

The dataset used in this study was obtained in 2016 from registers and administrative databases maintained by public authorities. The data were pseudonymized by Statistics Sweden and the authors did not have access to information that could identify individuals. Informed consent was therefore not required, nor was it feasible or ethically viable for the authors to obtain. The study was originally approved (no. 2012/238) by the Swedish Ethical Review Authority in June 2012, with an amendment specifying exact variables to include in the analysis approved in October 2015 (no. 2012/238/1), and an amendment concerning a change in project leader approved in May 2019 (no. 2019–02580).

## 5. Results

### 5.1 Local markets

To provide a better understanding of the four local market definitions, a brief description of their market areas is presented in Table 1. In terms of area, fixed radius markets of 1 km are the smallest (~3 km^2^), while the size of fixed radius markets of 3 km, with ~28 km^2^, is smaller than the average variable radius market (80 km^2^) and variable shape market (59 km^2^), but larger than the median variable radius and variable shape markets (14 km^2^ and 18 km^2^, respectively). In terms of radius, the variable radius markets (mean 3.3, SD 3.9, median 2.1) resemble the 3 km fixed radius. The large standard deviations for the variable radius and variable shape markets are affected by the occurrence of a few very large local markets located in more rural areas. Nine variable radius and three variable shape markets are larger than 1,000 km^2^. The largest variable radius market, located in a small town in northern rural Sweden, covers 11,471 km^2^.

**Table 1:** Summary statistics on size of markets for each local market definition.

	Fixed radius		Variable radius	Variable shape
	1 km	3 km		
**Area (km** ^**2**^ **)**	3	28		
Mean			80	59
SD			473	156
Median			14	18
**Population**				
Mean	7,752	36,337	19,470	22,556
SD	8,488	48,877	46,196	47,449
Median	5,085	17,437	10,412	11,605

In terms of population, fixed radius markets of 1 km are the smallest, with an average of 7,800 individuals (SD 8.5, median 5.1). This can be compared to mean values of 19,470–36,337 individuals for the other market definitions, which are also more similar to one another regarding standard deviations and median values. The size of the standard deviations from the three latter market definitions is affected by urban local markets covering large populations. The local market with the largest population for both the variable radius and variable shape definition, and third largest for the 3 km fixed radius definition, is derived from a provider located in Stockholm’s city centre. The provider’s local market covers an area of 245 km^2^ (variable radius) or 180 km^2^ (variable shape), with approximately 0.85 million people living within the market.

Two measures of competition are included in this study: the HHI and the number of providers. Regarding the HHI, values are highest (indicating lowest competition) for the fixed radius definition of 1 km (mean 0.79, SD 0.28), and lowest (indicating highest competition) for the fixed radius definition of 3 km (mean 0.54, SD 0.37). Variable radius markets and variable shape markets are very similar, with mean values of 0.69 (SD 0.33) and 0.66 (SD 0.34), respectively. Regarding the alternative competition measure, there is an average of two to five providers located in a local market. The number of providers within each local market is fairly similar for variable radius (mean 2.9, median 2) and variable shape markets (mean 3.1, median 2). For all four market definitions, a large share of local markets (35–60.9%, SD 0.28–0.37) is defined as monopolies (Table 2). For additional descriptive statistics and alternative operationalisations of local markets, see S1 Appendix.

**Table 2:** Summary statistics on competition levels for each local market definition.

	Fixed radius		Variable radius	Variable shape
	1 km	3 km		
**HHI**				
Mean	0.79	0.54	0.69	0.66
SD	0.28	0.37	0.33	0.34
Minimum value	0.15	0.04	0.02	0.02
Percentiles:				
10%	0.36	0.11	0.22	0.20
25%	0.51	0.20	0.36	0.36
50% (median)	1.00	0.42	0.77	0.63
75%	1.00	1.00	1.00	1.00
**Number of providers**				
Mean	1.8	4.7	2.9	3.1
SD	1.3	5.4	5.1	5
Median	1	3	2	2
Min–max	1–9	1–32	1–85	1–79
**Monopolies**				
Count	707	406	563	538
Share	60.9%	35.0%	48.5%	46.3%

Notes: Competition is measured both with HHI and as the number of providers located in each local market—lower HHI indicating higher competition and higher number of providers indicating higher competition. A local market is defined as a monopoly when HHI and number of providers equals 1.

**Fig 2:**
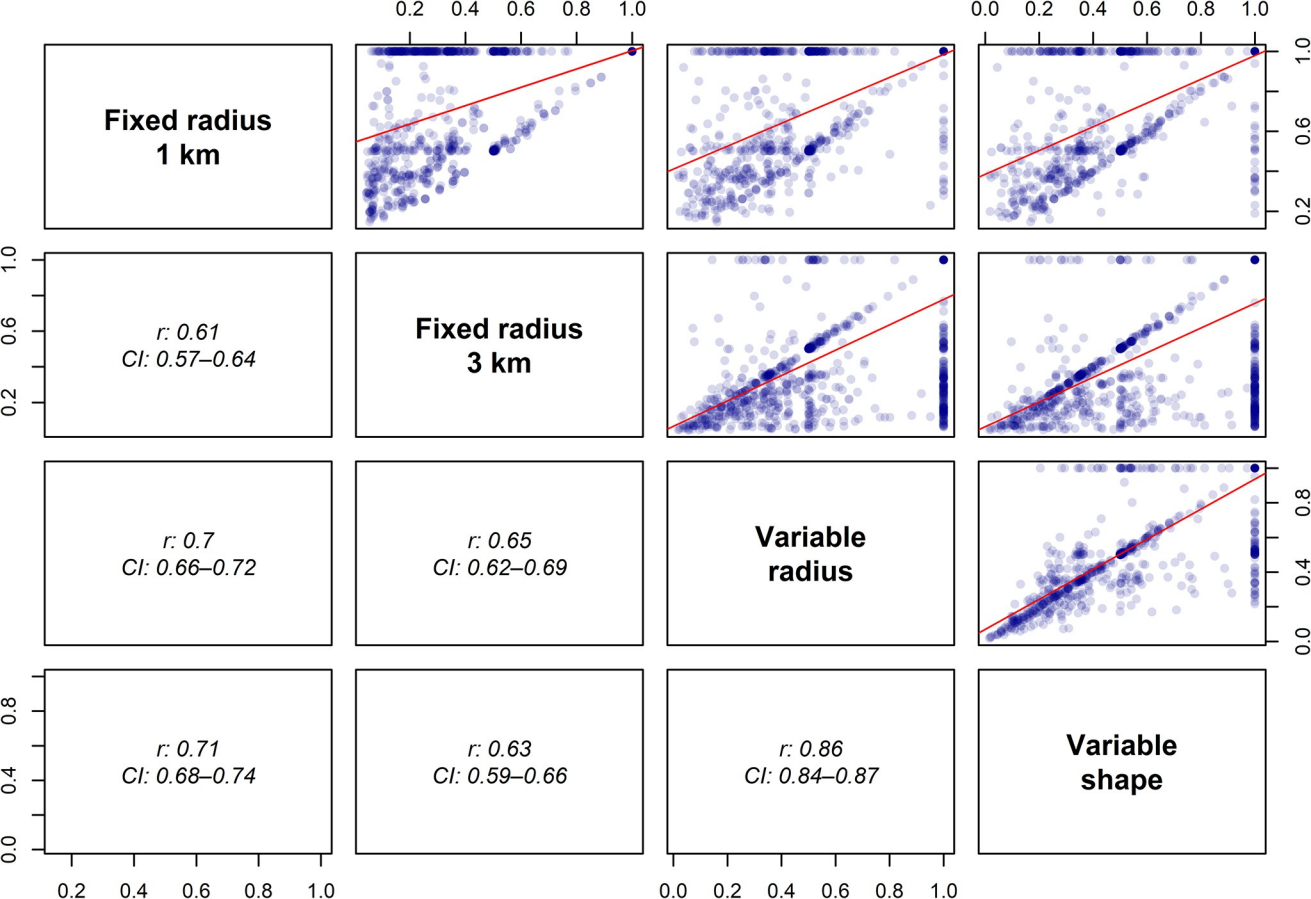
Correlation matrix. The figure shows a correlation matrix with correlation coefficients (Pearson’s r) and confidence intervals at the 95% level reported between each of the market definitions’ respective HHI measure.

The correlation coefficients for the HHI from the comparison of all four market definitions are presented in Fig 2. While the univariate descriptive statistics for variable radius and variable shape market definitions are similar (Table 2), they do not completely correlate (r 0.86, CI 0.85–0.88). As seen in the scatterplot with the variable radius and the variable shape definitions (at the bottom, to the right in Fig 2), they do not always define the same local markets as monopolies. The second highest correlation coefficients are found between fixed radius markets of 1 km and variable shape markets (r 0.71, CI 0.68–0.74), closely followed by fixed radius markets of 1 km and variable radius markets (r 0.70, CI 0.66–0.72). The fixed radius definition of 3 km correlates least with the other market definitions (r 0.61–0.65). Thus, we can see that differences exist between the HHI values based on the two variable market definitions, but based on Fig 2, we cannot determine which definition is best.

**Fig 3:**
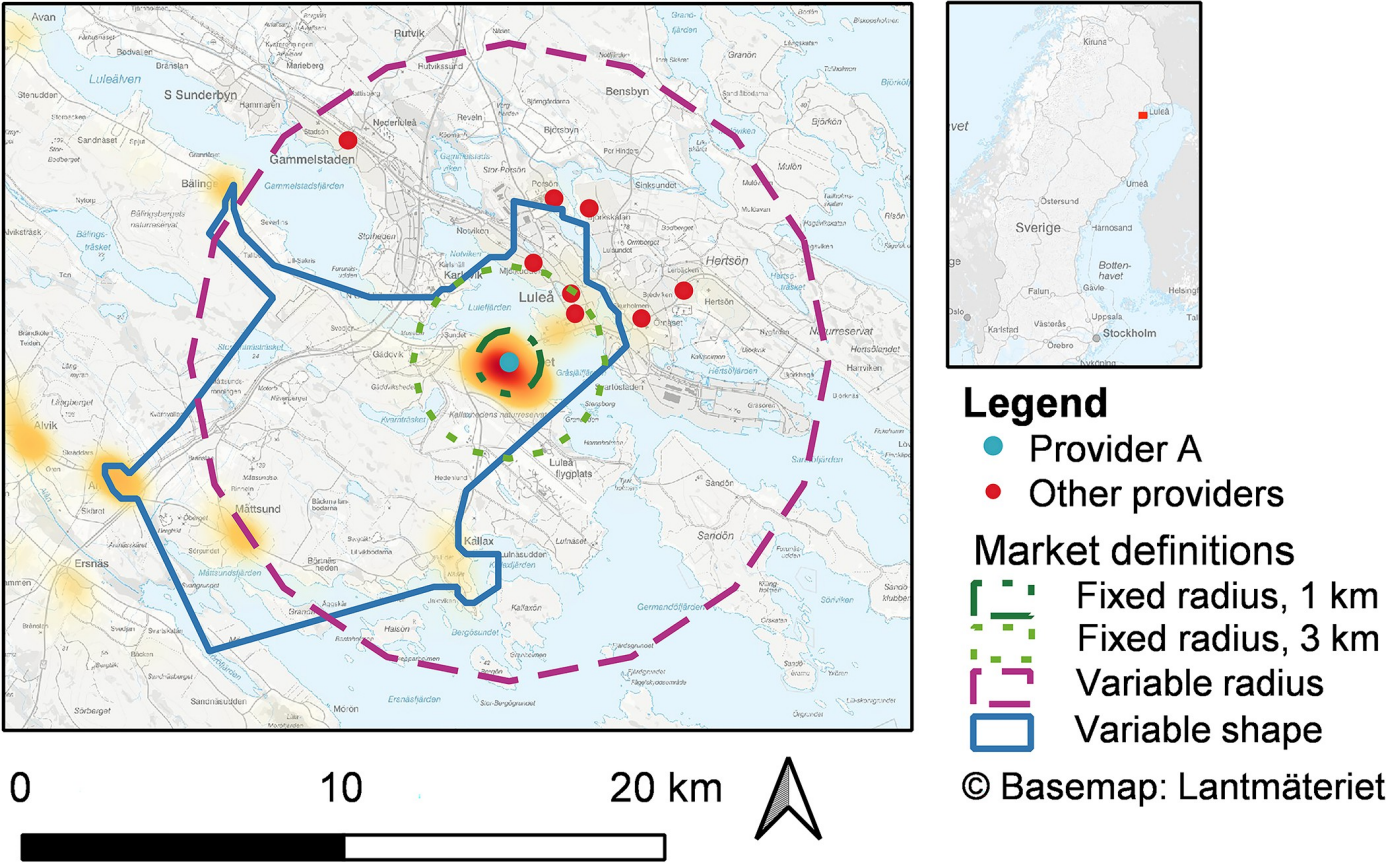
Local markets for Provider A. Heatmap of the Provider A’s (Bergnäset primary healthcare centre, Luleå) 80 per cent nearest registered patients with all four local markets definitions layered on top.

**Fig 4:**
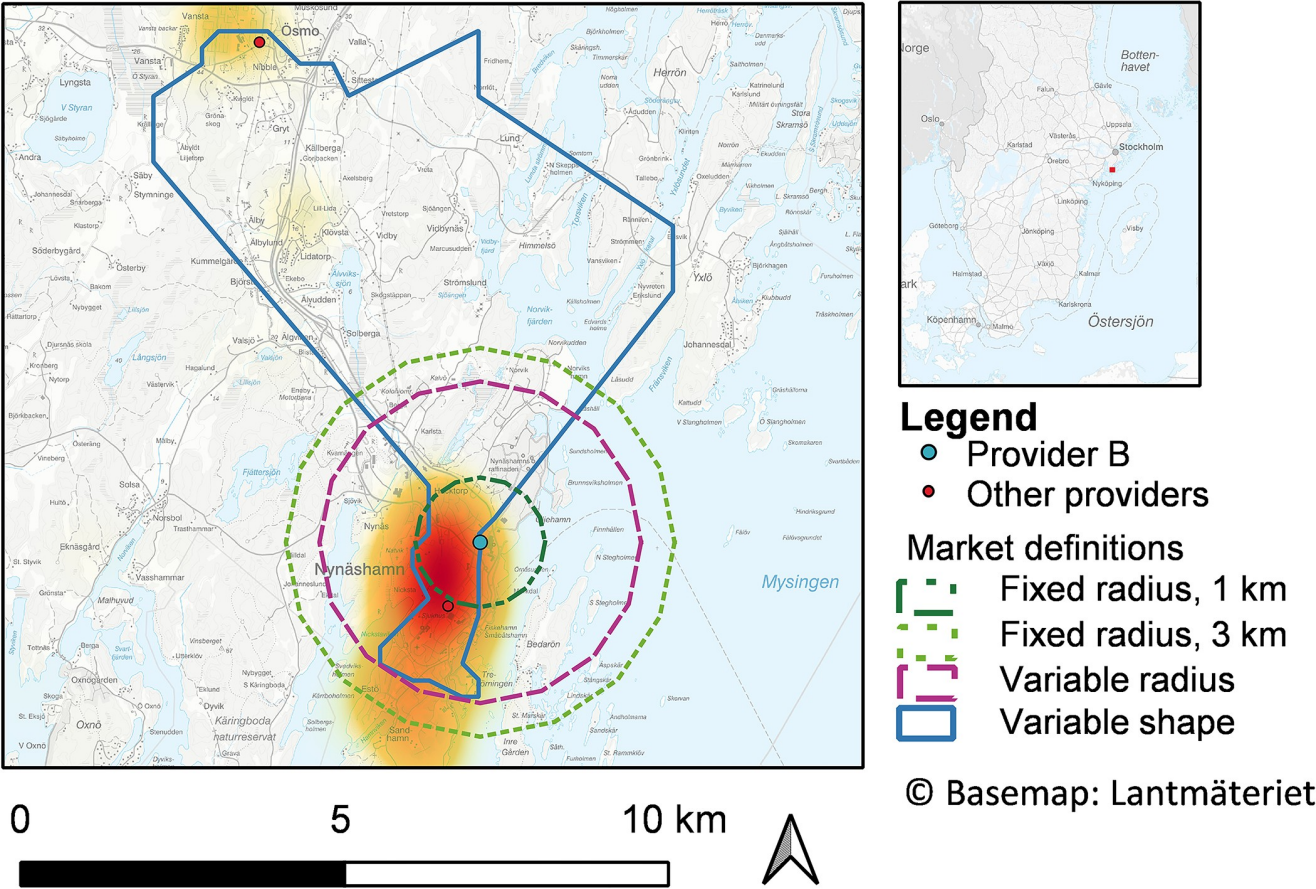
Local markets for Provider B. Heatmap of the Provider B’s (Nynäshamn primary healthcare centre, Nynäshamn) 80 percent nearest registered patients with all four local markets definitions layered on top.

For a visual illustration of how the different local market definitions capture the actual markets, heatmaps of two providers are presented in Figs 3 and 4, where the densities of individuals registered with the respective provider are shown. This illustrates the main catchment areas of each of the two providers, and thus where their actual market uptake is. Provider A (Bergnäset primary healthcare centre, Fig 3) is located near the town centre of Luleå. The highest densities of registered patients can be found in the town centre and residential areas nearby, but Provider A also attracts patients from more rural areas in a south-westerly direction, an area with many people commuting to the city centre and thereby passing by Provider A. The variable shape market is focused towards the southwest; providers on the other side of the city centre (in the northeast) are located outside the local market and are thus not treated as competitors to Provider A in the variable shape definition. These other providers are defined as competitors in the variable radius definitions, however.

For Provider B (Nynäshamn primary healthcare centre, Fig 4), the highest density of patients is found in the nearby town centre of Nynäshamn, in a south-easterly direction from Provider B, but the provider also attracts patients from more rural villages in a north-westerly direction. The variable shape market captures areas with the highest densities in central parts of the town as well as rural areas to the northwest, including another provider, which is treated as a competitor in the variable shape definition. This other provider is, however, not treated as a competitor to Provider B in the variable radius definition. The local market, as defined by the variable radius definition, instead captures larger parts of central Nynäshamn, where high densities of registered individuals are found.

To conclude, we find large variations in market area and market population both within and across local market definitions. For all four local market definitions, a significant share of markets is defined as monopolies. Regarding competition, the highest levels of competition are found in fixed radius markets of 3 km, while the lowest levels are observed in fixed radius market of 1 km. When comparing the four market definitions, it is noted that that 3 km radius markets correlate the least with the other market definitions, and that variable radius and variable shape markets are most correlated. Visual illustrations of two providers and their local markets and registered patients show how local markets can differ depending on definitions used. We now move on to the empirical examination of provider competition in Swedish primary care.

### 5.2 Competition in Swedish primary care

Competition levels vary significantly in Swedish primary care. A substantial part of the providers faces no competition at all (see Table 2), with 360 providers (31%) located in monopoly markets irrespective of the local market definition used. At the same time, providers located in competitive local markets face rather high levels of competition. When monopoly markets are removed, the HHI mean values drops substantially (i.e. competition level increases), from means between 0.54 and 0.79 (SD 0.28–0.37) to means between 0.29 and 0.47 (SD 0.16–0.19), depending on the local market definition used.

**Fig 5:**
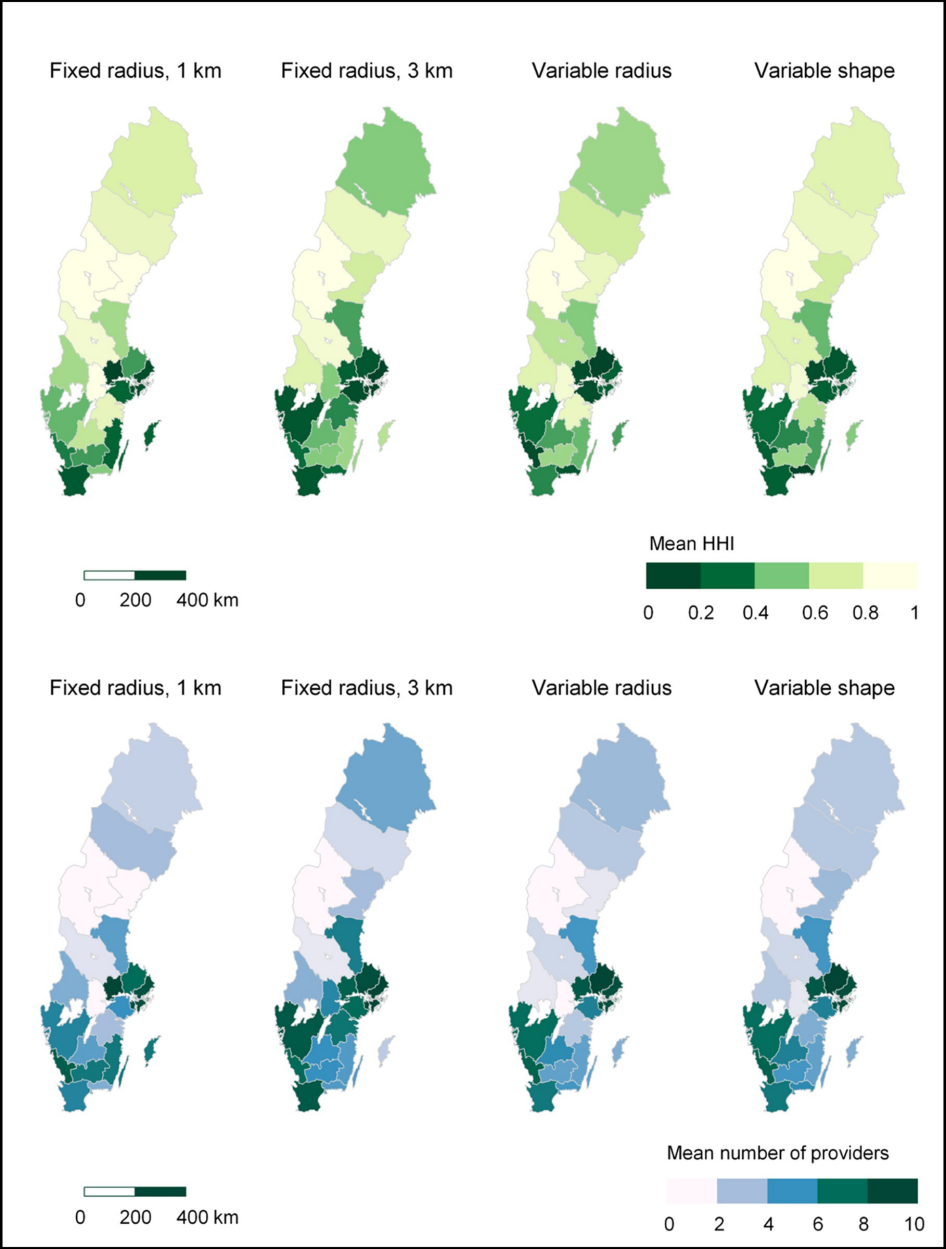
Mean level of competition in each region. Darker colours indicate higher competition. In the maps at the top, the mean HHI in each region is reported. In the maps at the bottom, competition is measured as the number of competitors; the mean number of providers located in the local markets in each region is reported. Source for basemap: Statistics Sweden.

**Fig 6:**
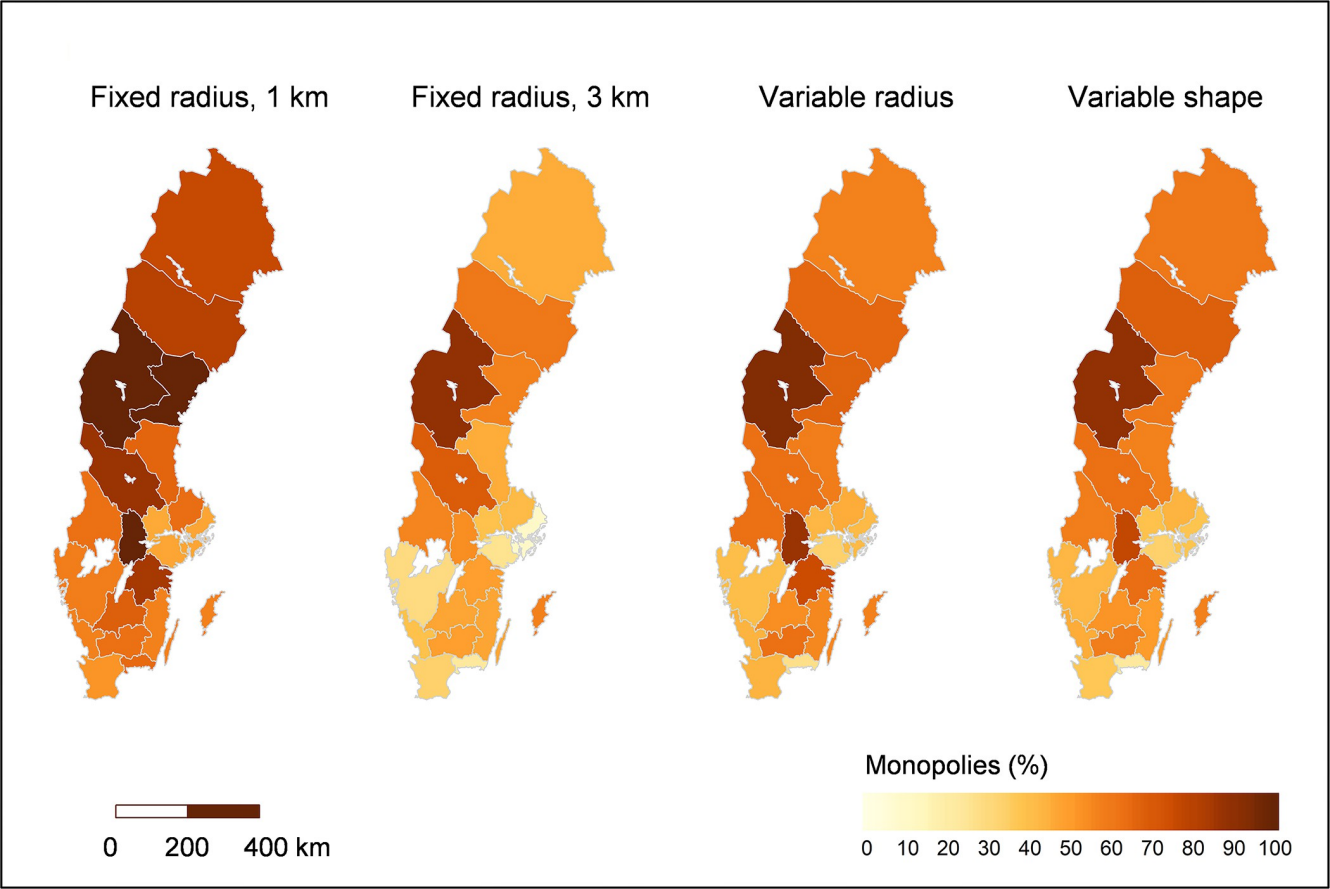
Share of local monopoly markets in the regions. Darker colours indicate higher share of monopoly markets in the region. Source for basemap: Statistics Sweden.

There is also a large variation in terms of where competitive local markets are found. Local markets with low HHI values (i.e. high competition) can be found across the country, not only in the largest cities. Looking at descriptive statistics aggregated to the regional level, however, the highest competition is found in regions with higher population density, while more rural regions tend to have a higher share of local markets facing low or no competition (see Fig 5). With the fixed radius definition of 1 km, all local markets in three of the 21 regions—Jämtland, Västernorrland and Örebro—are defined as monopoly markets (see Fig 6). For more regional statistics, see S2 Appendix.

**Fig 7:**
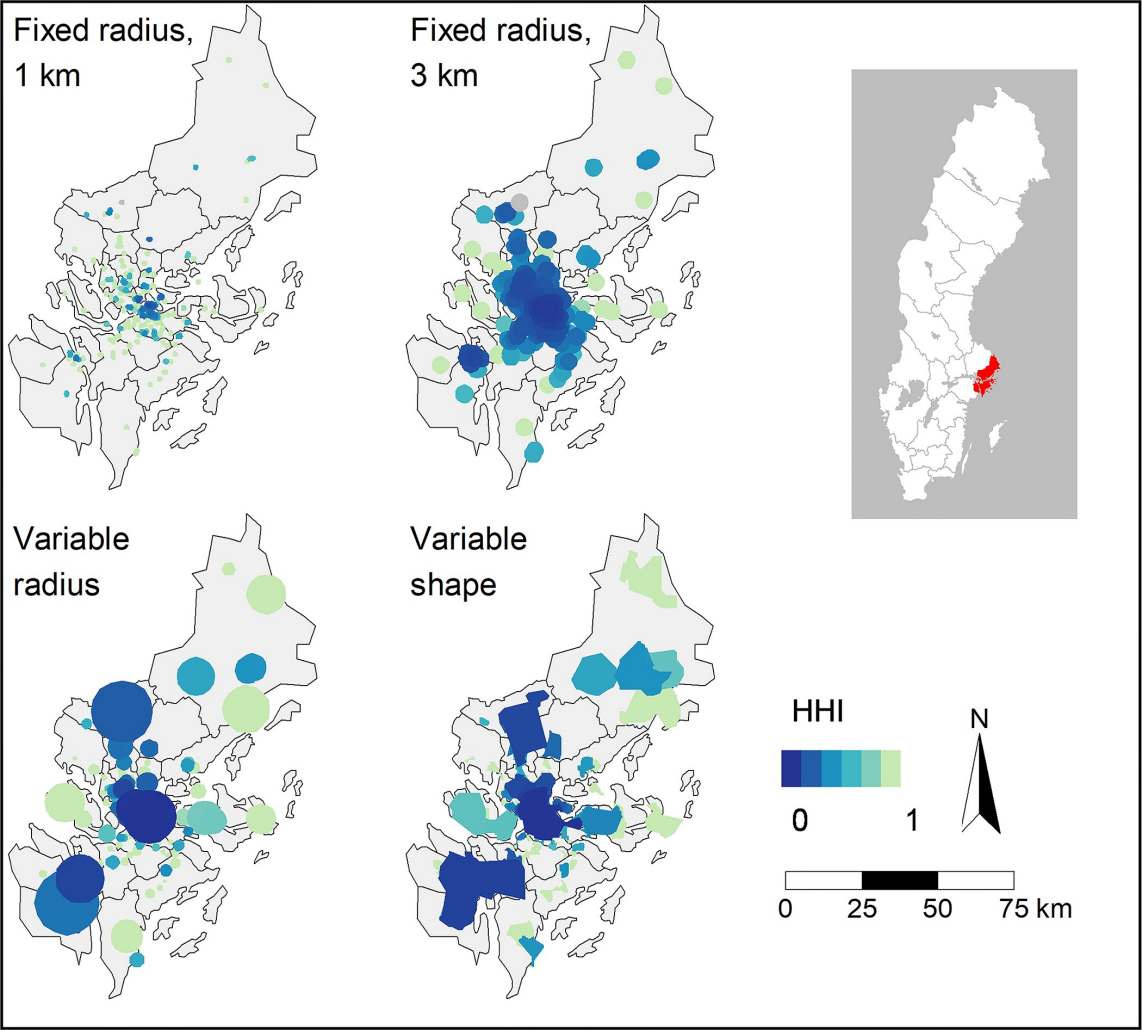
Local markets in Region Stockholm. HHI levels are indicated by colour. Darker colours indicate higher competition. Source for the basemap: Statistics Sweden.

**Fig 8:**
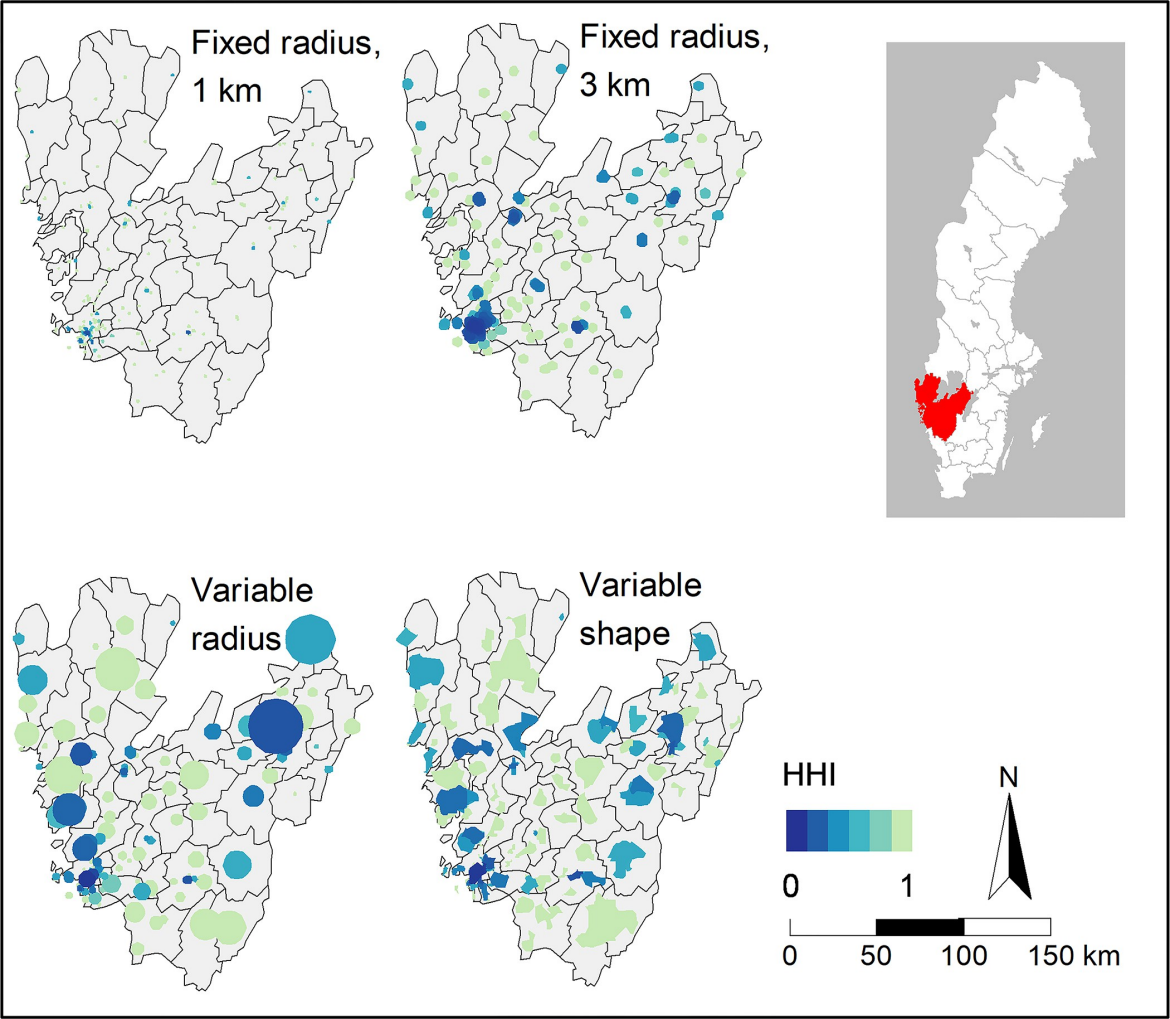
Local markets in Region Västra Götaland. HHI levels are indicated by colour. Darker colours indicate higher competition. Source for the basemap: Statistics Sweden.

The maps of Region Stockholm (Fig 7) and Region Västra Götaland (Fig 8) show local markets and their respective HHI value. Region Stockholm is both the most populous (~2.4 million) and the most densely populated Swedish region, and contains Stockholm, the capital and largest city. Region Västra Götaland is the second most populous (~1.7 million) and the third most densely populated region, and contains Sweden’s second-largest city, Gothenburg.

Competition levels vary within both regions. Even though a higher concentration of more competitive markets is found in central Stockholm and Gothenburg, highly competitive local markets are found across the regions. In Region Stockholm, the HHI is on average between 0.3 (fixed radius, 3 km) and 0.71 (fixed radius, 1 km), depending on local market definition. Even in Stockholm, the most densely populated region, 37–46 per cent of the local markets are defined as monopolies by three of the local market definitions, but only 8 per cent when defining markets by a fixed radius of 3 km. In Region Västra Götaland, the HHI is generally higher (i.e. lower competition) than in Stockholm, with an average HHI between 0.5 (fixed radius, 3 km) and 0.78 (fixed radius, 1 km). Between 28 and 58 per cent of the local markets are defined as monopolies in Region Västra Götaland. In both regions, less variation of competition is found in the city centres when fixed radius of 3 km is used to define local markets, compared to the other three market definitions. For more regional statistics, see S2 Appendix.

In sum, competition levels in the Swedish primary care market vary significantly—some providers face very high levels of competition, while approximately a third of the providers operate in monopoly markets. Geographically, variations are evident both within and across regions.

## 6. Discussion

In this paper, we set out to both contribute to the debate on local market definitions and examine competition in the Swedish primary care market. We begin by discussing the different local market definitions. Previous research has established that the choice of local market definition affects competition levels [58]. In this study, we find differences between the various local market definitions. In terms of population, the fixed radius definition of 1 km stands out. Only 7,752 people on average live in local markets defined by 1 km radiuses, which is much lower than both the variable radius and variable shape markets. The average number of registered patients per provider is 8,280, and a local market should, at least in most cases (an exception might be very remote providers), cover an area of more people than those registered with a provider. The fixed radius definition of 1 km likely leads to an urban-rural bias by creating markets that are too small for many providers, thereby over-predicting monopolies. The fixed radius definition of 3 km provides the least variation in competition within urban areas of Stockholm and Gothenburg, indicating that the market size in general is too large for urban areas. It also correlates less with the two variable market definitions than the fixed radius of 1 km does. This might be due to the large number of providers located in urban areas, for which both variable market definitions tend to generate local markets of smaller sizes, more comparable to fixed radius markets of 1 km than of 3 km. In sum, the fixed radius markets of 1 km fail to pick up on the variation across urban-rural areas, whereas fixed radius markets of 3 km fail to pick up on the variation within urban settings.

In countries like Sweden, where population densities vary significantly across the country, the urban-rural bias stemming from fixed radius markets is especially problematic, and there is a particular need for a variable market definition. While more computations are required for the variable shape definition than for the variable radius definition, the biggest computational difference is between the variable and fixed market definitions. Despite showing similar descriptive statistics, the two variable market definitions do not always define the same local markets as monopolies or include the same providers as competitors in a given local market. The heatmaps illustrating two providers and their registered patients indicate that the variable shape market definition might better capture areas with registered individuals and leave out areas without registered individuals further away from the provider. The variable radius market definition, however, might better capture areas nearby with high densities of residing patients. One possible refinement of the variable shape definition, to better capture nearby areas with a high density of registered patients, could be to relax the concavity of the polygons’ concave hulls. In addition, the sizes of both variable market definitions could be increased by choosing other percentiles. Determining which of the two variable market definitions most accurately captures the actual local markets is challenging, as they need to be compared against a benchmark. Further analyses are therefore needed, and we welcome future studies to examine the variable shape definition.

Moving on to the empirical examination of Swedish primary care, we find that a substantial portion of providers (35–61%) experience no competition at all, with a third of the providers being located in local markets defined as monopolies in all local market definitions. This is in line with findings from a study of English hospital markets, where the authors concluded that using fixed and variable radiuses results in a large part of local markets being described as perfect monopolies [58].

The proportion of monopolies is not only affected by the chosen local market definition, however, but also by how competitors are defined. In this study, all providers located within a specific local market are defined as competitors in that local market. Another way to identify competitors is to include providers whose local markets overlap [e.g. 13, 22]. The ratio of monopolies that most accurately depicts Swedish primary care can be debated, and how competitors are best defined can, and should, be further discussed. However, when operationalising competitors as those with overlapping markets (see S1 Appendix), about a fifth of the local markets are still defined as monopolies. The occurrence of monopolies has implications for whether competition can enhance quality (which economic theory states it will under fixed prices) and for the possibility of patients choosing a provider. In a substantial part of Swedish primary care, the preconditions for this to occur are lacking. While there might still be some competitive pressure on providers in local markets defined as monopolies (e.g., through the threat of competition), one of policymakers’ main intentions with the Patient Choice reform—i.e. to improve quality through provider competition—seems to fall short at the first hurdle: a competitive market structure. It should be noted, however, that despite lacking competition, a provider can still deliver high quality services, and policymakers can use other strategies, besides competition, to strengthen primary care, such as financial incentives, national workforce strategies, resources to professional quality improvement, or benchmarking [59].

Swedish primary care providers that are exposed to competition, however, face fairly high levels of it. Urban areas generally provide more favourable conditions for competition, providing larger patient bases and being more economically viable for private providers [60]. We find that while high competition is found in larger cities (at least in the cases of Region Stockholm and Region Västra Götaland), it is not only providers located in the largest cities or regions that experience competition. This is in line with a study of the English GP market, which found competition generally to be higher in urban areas but also that there is a large variation within both urban and rural areas [6]. We have not conducted thorough analysis of the geographical distribution of competition, however. Further research is needed to capture fully the urban-rural dimension of competition in the Swedish primary care market.

In this study, the mean values for HHI range between 0.54 and 0.79. As points of reference, GP competition has been found to be much higher in the US (HHI mean: 0.13) and slightly higher in Belgium (HHI mean: 0.31) [22, 24], whereas lower competition (HHI means: 0.62–0.85) was found in a study of hospital competition in England [58]. It is hard to find a good yardstick, and comparisons across contexts are difficult. Belgium, for example, is geographically smaller than Sweden, with less diversity in urban-rural population density, and its primary care system with small-sized GP practices means lower entry costs than in the Swedish primary care market. With more favourable competition conditions in Belgium, higher competition is to be expected than in Sweden. Nor is it surprising that higher competition is found in Swedish primary care than among English hospitals, as Swedish providers are of considerably smaller size than English hospitals. At the same time, Sweden has a lower population density and larger rural and remote areas than England, which may imply a larger share of geographically remote providers, and people are generally willing to travel longer distances for hospital care than for primary care [61].

We have examined the market structure of Swedish primary care, but not if and how provider competition impacts different performance dimensions. In a Swedish context, Dietrichson et al. [25] found small effects of competition on patients’ satisfaction with quality of care, but no effects on avoidable hospitalisations or patients’ satisfaction with access. The unit of analysis was, however, the municipality, not the provider. Therefore, it cannot be ruled out that the results are affected by other municipality-level factors. The literature on primary care competition is growing, but it remains limited. In addition, primary care systems are diverse, making it difficult to generalise findings from, for example, primary care sectors dominated by GPs running their own or group practices to Swedish primary care with its large multidisciplinary healthcare centres. More studies are therefore needed on primary care competition under fixed prices in general, and in particular on competition among larger primary care units, such as Swedish primary care providers.

### 6.1 Policy implications

This is the first study where competition among primary care providers in Sweden has been measured and analysed. This is noteworthy, considering that provider competition, and its anticipated positive effects, was one of the policy objectives of the Patient Choice reform more than a decade ago. The fact that patient registration data must be retrieved from each region complicates the study of competition in Swedish primary care. Nevertheless, Sweden has rich population databases—containing, for example, coordinate data on residence and personal identification number—enabling complete data analyses. Therefore, we encourage policymakers in the future to better enable thorough evaluations of reforms such as the Patient Choice reform.

The large share of monopolies and the variation of competition among Swedish providers indicate varying preconditions for competition to affect market performance. Based on our findings, policymakers should decide whether to continue pursuing competition—and, if so, how it could be strengthened in areas with low levels of competition. Strategies for increasing the supply of providers in targeted areas involve financially rewarding providers located in under-supplied areas and restricting new providers from establishing in saturated areas [62]. Regarding the first, many regional reimbursement systems already include additional payments to providers located in rural areas and following legislative changes in 2022, regions can now have separate patient choice systems (encompassing provider requirements and reimbursement systems) for different geographical areas [50]. The latter imposes restrictions on the freedom of establishment and has so far not been implemented. Both strategies involve further market regulations and which way to proceed is a political decision that involves weighing different values and interests against each other. Competition is also not the only policy objective in Swedish healthcare, neither as stated in the Patient Choice reform [45] nor in the Health and Medical Services Act [51], and other objectives can be pursued, irrespective of market ambitions. Policymakers could therefore choose to focus on other ways than competition to both foster quality and achieve other policy objectives, such as accessibility and equity—ways that might or might not contradict the principles of competition.

### 6.2 Methodological considerations

A few methodological limitations should be addressed. First, an unknown number of individuals in our data have been automatically registered with a provider by the region in the absence of an active, individual choice of provider. Automatic registration does not reflect patients’ own choice, and can therefore be problematic for our study. Since we do not know how many, or which, individuals have been automatically registered with a provider, we unfortunately cannot undertake any further analyses on providers’ local markets as reflected solely by the residence of patients who made an active choice. However, since the regions register individuals with a nearby provider and proximity is a key predictor of choice, it is unclear whether this has affected our results.

Second, there are potential inaccuracies in the patient registration data. As most people in Sweden are registered with a provider irrespective of whether they use primary healthcare services, people who do not frequently use primary healthcare services might not consider changing provider when moving. Therefore, local market areas pertaining to the two variable market definitions might, to some extent, be based on data not representing the actual markets. Theoretically, one solution could be to use patient visit data instead of patient registration data. However, this would require data covering a long time period to capture less frequent patients.

Third, the geographic boundaries of local markets are always, to some degree, subject to arbitrary decisions in terms of both distances for fixed radius markets and percentile distances for variable markets. We have dealt with this issue by providing data on competition measures based on alternative local market definitions in S1 Appendix.

## 7. Conclusions

Decisions on which local market definition to use are important as they affect how competition in local markets is described. Fixed radius market definitions fail to pick up on the variation both across urban-rural areas and within urban areas. This is problematic in countries such as Sweden, with highly diverging population densities across the country. Variable radius and variable shape definitions better capture the actual markets. Despite showing similar descriptive statistics, the two variable market definitions sometimes define different providers as competitors in a local market, and there are several instances where a local market is defined as a monopoly by one variable market definition but not the other. The variable shape market seems to better capture geographical areas where registered patients live and thus define competitors more accurately. Further analyses are needed, however, and we welcome future studies to examine the variable shape definition.

We have shown that competition in Swedish primary varies significantly, especially using the variable shape and variable radius definitions. A substantial portion of the providers experiences no competition at all. At the same time, providers exposed to competition in general face rather high levels of it. Further analyses of the Swedish primary care market are needed, and policymakers should decide whether competition should still be pursued, and, if so, how competition could be strengthened in monopoly markets.

## Supporting information

S1 Appendix Additional summary statistics. (DOCX)

S2 Appendix Summary statistics, aggregated to regional level. (DOCX)

S1 Data Provider-level data. (CSV)
