# Supplementary material for: Measuring competition in primary care—Evidence from Sweden
Source: PLoS One. 2024 Jul 15;19(7):e0304994. doi: 10.1371/journal.pone.0304994 (PMC11249268; doi:10.1371/journal.pone.0304994)
Supplement: S1 Appendix — (DOCX) [file pone.0304994.s001.docx]

# S1 Appendix. Additional summary statistics

In S1 Appendix, additional data are presented to enable a deeper understanding of the market structure of Swedish primary care and the different market definitions. In Table 1, descriptive statistics pertaining to HHI are presented for eight local market definitions. Fixed radius 2 km and 4 km, variable radius based on percentile 60, and variable shape based on percentile 80 (step 1) and percentile 75 in each segment (step 2) are added to the list of market definitions presented in the paper.

| **Table 1.** Summary statistics for eight local markets definitions | | | | | | | | | | |
| --- | --- | --- | --- | --- | --- | --- | --- | --- | --- | --- |
|  | **Fixed radius** | | | | | **Variable radius** | | **Variable shape** | | |
|  | 1 km | 2 km | 3 km | 4 km | 55* | | 60* | 80-70** | 80-75** | |
| Mean | 0.79 | 0.62 | 0.54 | 0.49 | 0.69 | | 0.65 | 0.66 | 0.64 | |
| SD | 0.28 | 0.34 | 0.37 | 0.38 | 0.33 | | 0.34 | 0.34 | 0.34 | |
| Min | 0.15 | 0.07 | 0.04 | 0.03 | 0.02 | | 0.01 | 0.02 | 0.02 | |
| Percentiles |  |  |  |  |  | |  |  |  | |
| 10% | 0.36 | 0.19 | 0.11 | 0.08 | 0.22 | | 0.19 | 0.2 | 0.18 | |
| 25% | 0.51 | 0.31 | 0.2 | 0.15 | 0.36 | | 0.34 | 0.36 | 0.34 | |
| 50% | 1 | 0.54 | 0.42 | 0.35 | 0.77 | | 0.6 | 0.63 | 0.57 | |
| 75% | 1 | 1 | 1 | 1 | 1 | | 1 | 1 | 1 | |
| Monopolies | 707 (60.6%) | 470 (40.5%) | 405 (34.9%) | 370 (31.9%) | 564  (48.6%) | | 513  (44.2%) | 537  (46.3%) | 513  (44.2%) | |
| * The percentile distance between a PHCC and registered individuals, used as the radius of the local markets.  ** The first number refers to the percentile distance used in the first step to capture the nearest registered individuals. The second number refers to the percentile distance used in the second step to capture the nearest registered individual, of those defined in step one, within each of the eight distance direction categories. | | | | | | | | | |  |

In Table 2, descriptive statistics are presented on the same market definitions as in Table 1. However, here, instead of defining competitors as the PHCCs located in a local market, they are defined as PHCCs whose local markets overlap, resulting in lower HHI and fewer monopolies.

| **Table 2.** HHI with competitors defined as those with overlapping markets | | | | | | | | |
| --- | --- | --- | --- | --- | --- | --- | --- | --- |
|  | **Fixed radius** | | | | **Variable radius** | | **Variable shape** | |
|  | 1 km | 2 km | 3 km | 4 km | 55* | 60* | 80-70** | 80-75** |
| Mean | 0.67 | 0.54 | 0.48 | 0.45 | 0.56 | 0.52 | 0.5 | 0.67 |
| Diff. with mean  in Table 1 | (-0.12) | (-0.08) | (-0.06) | (-0.04) | (-0.13) | (-0.13) | (-0.11) | (0.03) |
| SD | 0.31 | 0.36 | 0.37 | 0.37 | 0.33 | 0.33 | 0.32 | 0.31 |
| Min | 0.1 | 0.05 | 0.04 | 0.03 | 0.02 | 0.01 | 0.02 | 0.1 |
| Percentiles |  |  |  |  |  |  |  |  |
| 10% | 0.24 | 0.13 | 0.08 | 0.06 | 0.16 | 0.14 | 0.13 | 0.24 |
| 25% | 0.38 | 0.21 | 0.15 | 0.12 | 0.27 | 0.24 | 0.22 | 0.38 |
| 50% | 0.64 | 0.41 | 0.35 | 0.31 | 0.5 | 0.45 | 0.41 | 0.64 |
| 75% | 1 | 1 | 1 | 0.96 | 1 | 0.91 | 0.83 | 1 |
| Monopolies | 1 | 1 | 1 | 1 | 1 | 1 | 1 | 1 |
| * The percentile distance between a PHCC and registered individuals, used as the radius of the local markets. | | | | | | | | |
| ** The first number refers to the percentile distance used in the first step to capture the nearest registered individual. The second number refers the to the percentile distance used in the second step to capture the nearest registered individual, of those defined in step one, within each of the eight distance direction categories. | | | | | | | | |

| **Table 3.** Correlation coefficients, comparisons of HHI based on the different definitions of competitors used in Table 1 and 2 | | | | |
| --- | --- | --- | --- | --- |
|  | Pearson’s *r* | CI 95 % | | N |
|  |  | Low | High |  |
|  |  |  |  |  |
| **Fixed radius** |  |  |  |  |
| 1 km | 0.79 | 0.77 | 0.81 | 1160 |
| 2 km | 0.90 | 0.89 | 0.91 | 1160 |
| 3 km | 0.94 | 0.93 | 0.95 | 1160 |
| 4 km | 0.96 | 0.95 | 0.96 | 1160 |
|  | |  |  |  |
| **Variable radius** | |  |  |  |
| 55^th^ percentile | 0.82 | 0.80 | 0.84 | 1161 |
| 60^th^ percentile | 0.83 | 0.81 | 0.84 | 1161 |
|  | |  |  |  |
| **Variable shape** | |  |  |  |
| 80-70 percentile | 0.80 | 0.77 | 0.82 | 1161 |
| 80-75 percentile | 0.81 | 0.79 | 0.83 | 1161 |
|  |  |  |  |  |
| *Note:* Comparisons of HHI for local markets based on competitors defined as providers located within a market (see Table 1) and HHI based on competitors defined as providers with overlapping markets (see Table 2). | | | | |
