# Supplementary material for: Measuring competition in primary care—Evidence from Sweden
Source: PLoS One. 2024 Jul 15;19(7):e0304994. doi: 10.1371/journal.pone.0304994 (PMC11249268; doi:10.1371/journal.pone.0304994)
Supplement: S2 Appendix — (DOCX) [file pone.0304994.s002.docx]

# **S2 Appendix.** Summary statistics, aggregated to regional level

| **Table 1.** Descriptive statistics for HHI, per region and market definition | | | | | | | | | |
| --- | --- | --- | --- | --- | --- | --- | --- | --- | --- |
| **Region** |  | **Fixed radius,**  **1 km** | | **Fixed radius,**  **3 km** | | **Variable**  **radius** | | **Variable**  **shape** | |
|  | n | Mean  (SD)  *Median* | Monopolies  (%) | Mean  (SD)  *Median* | Monopolies  (%) | Mean  (SD)  *Median* | Monopolies  (%) | Mean  (SD)  *Median* | Monopolies  (%) |
| Blekinge | 19 | 0.81  (0.26)  *1* | 63 % | 0.55  (0.25)  *0.47* | 21% | 0.6  (0.26)  *0.54* | 26% | 0.54  (0.27)  *0.38* | 21% |
| Dalarna | 28 | 0.93  (0.17)  *1* | 86% | 0.81  (0.28)  *1* | 68% | 0.77  (0.3)  *1* | 61% | 0.79  (0.27)  *1* | 61% |
| Gotland | 7 | 0.75  (0.31)  *1* | 57% | 0.72  (0.35)  *1* | 57% | 0.7  (0.38)  *1* | 57% | 0.72  (0.35)  *1* | 57% |
| Gävleborg | 43 | 0.82  (0.26)  *1* | 65% | 0.63  (0.36)  *0.58* | 44% | 0.73  (0.34)  *1* | 58% | 0.71  (0.34)  *1* | 56% |
| Halland | 48 | 0.76  (0.3)  *1* | 56% | 0.55  (0.36)  *0.34* | 38% | 0.59  (0.36)  *0.43* | 42% | 0.6  (0.37)  *0.43* | 44% |
| Jämtland | 25 | 1  (0)  *1* | 100% | 0.94  (0.17)  *1* | 88% | 0.95  (0.17)  *1* | 92% | 0.94  (0.16)  *1* | 88% |
| Jönköping | 46 | 0.84  (0.25)  *1* | 67% | 0.65  (0.35)  *0.64* | 46% | 0.7  (0.34)  *1* | 52% | 0.69  (0.34)  *0.85* | 50% |
| Kalmar | 37 | 0.75  (0.3)  1 | 57% | 0.67  (0.32)  *0.59* | 46% | 0.72  (0.33)  *1* | 54% | 0.7  (0.32)  *0.6* | 49% |
| Kronoberg | 31 | 0.77  (0.31)  *1* | 61% | 0.66  (0.36)  *0.6* | 48% | 0.75  (0.33)  *1* | 61% | 0.73  (0.34)  *1* | 58% |
| Norrbotten | 32 | 0.89  (0.2)  *1* | 75% | 0.66  (0.32)  *0.61* | 44% | 0.75  (0.31)  *1* | 56% | 0.79  (0.27)  *1* | 59% |
| Skåne | 151 | 0.74  (0.27)  *1* | 50% | 0.5  (0.37)  *0.36* | 32% | 0.65  (0.32)  *0.53* | 42% | 0.6  (0.33)  *0.52* | 36% |
| Stockholm | 214 | 0.71  *0.77*  (0.3) | 46% | 0.3  (0.26)  *0.2* | 8% | 0.63  (0.34)  *0.59* | 40% | 0.6  (0.35)  *0.51* | 37% |
| Södermanland | 28 | 0.75  (0.27)  *0.89* | 46% | 0.48  (0.32)  *0.35* | 25% | 0.59  (0.31)  *0.5* | 32% | 0.6  (0.3)  *0.46* | 32% |
| Uppsala | 48 | 0.77  (0.31)  *1* | 62% | 0.5  (0.42)  *0.23* | 40% | 0.57  (0.4)  *0.5* | 44% | 0.57  (0.4)  *0.51* | 42% |
| Värmland | 31 | 0.82  (0.23)  *1* | 61% | 0.75  (0.31)  *1* | 55% | 0.8  (0.27)  *1* | 61% | 0.79  (0.27)  *1* | 58% |
| Västerbotten | 39 | 0.9  (0.2)  *1* | 79% | 0.76  (0.3)  *1* | 59% | 0.79  (0.3)  *1* | 64% | 0.81  (0.28)  *1* | 67% |
| Västernorrland | 32 | 1  (0)  *1* | 100% | 0.74  (0.31)  *1* | 56% | 0.83  (0.25)  *1* | 66% | 0.77  (0.29)  *1* | 59% |

| **Table 1** continued: | | | | | | | | | | |
| --- | --- | --- | --- | --- | --- | --- | --- | --- | --- | --- |
| **Region** |  | **Fixed radius,**  **1 km** | | **Fixed radius,**  **3 km** | | **Variable**  **radius** | | **Variable**  **shape** | |  |
|  | n | Mean  (SD)  *Median* | Monopolies  (%) | Mean  (SD)  *Median* | Monopolies  (%) | Mean  (SD)  *Median* | Monopolies  (%) | Mean  (SD)  *Median* | Monopolies  (%) |  |
| Västmanland | 29 | 0.65  (0.34)  *0.52* | 45% | 0.53  (0.4)  *0.5* | 38% | 0.59  (0.38)  *0.51* | 41% | 0.56  (0.38)  *0.5* | 38% |  |
| Västra  Götaland | 201 | 0.78  (0.28)  *1* | 58% | 0.5  (0.36)  *0.36* | 28% | 0.64  (0.32)  *0.57* | 39% | 0.64  (0.33)  *0.55* | 41% |  |
| Örebro | 29 | 1  (0)  *1* | 100% | 0.66  (0.37)  *1* | 52% | 0.93  (0.19)  *1* | 86% | 0.85  (0.27)  *1* | 76% |  |
| Östergötland | 42 | 0.9  (0.23)  *1* | 83% | 0.6  (0.39)  *0.52* | 48% | 0.83  (0.3)  *1* | 74% | 0.76  (0.32)  *1* | 62% |  |
